# MicroRNAs 9 and 370 Association with Biochemical Markers in T2D and CAD Complication of T2D

**DOI:** 10.1371/journal.pone.0126957

**Published:** 2015-05-15

**Authors:** Tarek M. Motawae, Manal F. Ismail, Marwa I. Shabayek, Mae M. Seleem

**Affiliations:** 1 Department of Biochemistry, Faculty of Pharmacy, Cairo University, Cairo, Egypt; 2 Department of Biochemistry, Faculty of Pharmaceutical Sciences and Pharmaceutical Industries, Future University, Cairo, Egypt; SAINT LOUIS UNIVERSITY, UNITED STATES

## Abstract

**Background:**

MicroRNAs (miRNAs) are small non coding RNAs with essential roles, of which any alteration leads to several conditions. Their roles in diabetes (DM) and its vascular complications have not been completely assessed.

**Aim:**

to study the association of two miRNAs; 9 and 370, with biochemical parameters of type 2 diabetic (T2D), dyslipidemia and coronary artery disease (CAD).

**Subjects and Methods:**

Blood samples were taken from 200 subjects of both genders, in the Outpatient clinic of Al Qasr El-Einy teaching hospitals, in which levels of both miRNAs (using real time PCR) and routine parameters were measured. Subjects were divided over four groups, 50 in each group as follows; patients with T2D, patients with CAD, patients with T2D and CAD, and healthy control subjects.

**Main Outcome:**

miRNA 9 levels were expected to be over expressed in diabetic patients, while miRNA 370 levels were expected to be over expressed in those suffering from CAD and their association with CAD complication of T2D.

**Results:**

miRNA 9 levels were significantly higher in T2D patients and T2D patients with CAD, (1.18±0.07, and 1.31±0.08 respectively), while miRNA 370 levels were significantly higher in T2D patients, CAD patients, and T2D patients with CAD (0.59±0.05, 1.00±0.05, and 1.20±0.06 respectively), compared to control group at p = 0.000. In addition both miRNAs were still significantly associated with each other even after conducting multiple regression analysis.

**Conclusion:**

This study associates the possible role of miRNAs in the diagnosis/prognosis of CAD complication of T2D.

## Introduction

One the most common complication of diabetes mellitus (DM) is coronary artery disease (CAD), an estimate of two thirds of deaths in DM is attributed to its cardiovascular complication [1].

Pathogenesis of vascular complications in diabetic patients is attributed to hyperglycemia-induced inflammation [2]. Vasoconstriction occurs due to increased synthesis of vasoconstrictors, together with decreased production of vasodilators [3]. All lead to oxidative stress, both micro and macro-vascular cellular damage, and finally arteriolosclerosis [4]. This results in the development of metabolic syndrome that is an accumulation of cardiovascular risk factors; obesity, hyperglycemia, increased blood pressure, type 2 diabetes (T2D), and hyperlipidemia [4].

MicroRNAs (miRNAs) are a group of small, non coding RNAs which regulate expression of complementary mRNAs. By directly regulating protein translation processes, miRNAs are considered to be regulators of almost biological processes. Alterations in regulation of miRNAs have been associated with many clinical conditions, especially CAD [5], as miRNAs have been proved to be associated with the incidence of cardiac hypertrophy [6], fibrosis [7], angiogenesis [8], and heart failure (HF) [9].

In addition, miRNAs are important for the pancreatic development, particularly β-cells. Conditional removal of Dicer [10], an essential cleavage protein in miRNA synthesis, that results in complete alteration of miRNA transcripts in progenitor pancreatic cells producing significant changes in the final pancreatic cell lines. Most of the genes expressed by β cells of the pancreas are regulated in accordance with alterations in blood glucose thus optimizing insulin production and secretion [11]. Release, transcription, stability, and translation of insulin and insulin messenger RNA are controlled by the concentration of glucose in pancreas [12]. The need for more profound studies relating miRNA to DM still remains, however it is obvious that mutations or alteration of miRNA can result in pancreatic pathologies especially those involving β cells.

MicroRNA 9 is one of the miRNAs involved in β cells pathologies, as it is expressed in these cells. MicroRNA 9 increased expression lowers the glucose-stimulated secretion of insulin [13].

In addition to DM, an in-vivo study demonstrated that infusions of miRNA9 analogs decreased cardiac hypertrophy caused by isoproterenol thus improving the overall heart function, by regulating myocardin which is targeted by miRNA 9 [14].

Since the 1990s miRNAs have been proved to be involved in almost all metabolic and cellular processes. Several evidence showed that alteration in lipid metabolism regulation can lead to metabolic associated diseases [15]. Beside the known regulators of transcription, sterol regulatory element-binding proteins (SREBPs) and liver X receptors (LXRs), miRNAs have an essential role in regulating post-transcriptionally essential genes associated with lipid regulation, one of these miRNAs is miRNA370 [16]. Evidence from several studies have showed that several miRNAs, especially miRNA 370, have essential roles in regulating lipid metabolism [17]. In case of CAD, miRNA 370 showed positive association with dyslipidemia including total cholesterol (TC), triacyglycerol (TAG), and low density lipoprotein-cholesterol (LDLc), all of which can lead to the development of CAD[16].

The current study was carried out to assess the changes in the sera levels of miRNAs 9 and 370 in T2D subjects, CAD subjects, and subjects suffering from both compared to healthy control ones. In addition, correlation between both miRNAs and other T2D associated hyperglycemia and dyslipidemia biochemical parameters was carried out in order to find out how these possible markers are associated with the incidence and pathogenesis of CAD on top of T2D.

## Subjects and Methods

### 2.1 Subjects

This study was approved by the committee of Medical ethics of Cairo University and an informed consent was signed and obtained from each subject. All regulations and recommendations of the declaration of Helsinki were followed. Blood was withdrawn from two hundred subjects of both genders in the Outpatient clinic of Al-Qasr Al-Einy Teaching hospitals from patients and healthy relatives aging from 50 to 68 years. The groups were classified as follows: Group I included 50 healthy non-diabetic control subjects, group II included 50 patients with T2D, group III included 50 patients with CAD, and group IV included 50 patients with T2D and CAD. CAD (whether: angina, clots and ischemia) was diagnosed by either laboratory analysis, or angiogram. Body mass index (BMI) was calculated by dividing the weight (in kilograms) by the square of the height (in meters), and calculated on the same day of sample withdrawal. Subjects included both age matched genders; males and females. The effect of subject sex was not taken into consideration in our analyses as all subjects had the same heart disease risk (all females were postmenopausal). Characteristics of the current study subjects are shown in S1 Table.

Medical history was obtained from each subject; duration of T2D (not less than 10 years), diabetes control (all T2D had controlled HbA_1_c between 11 to 12.5%), presence of familial DM, type of CAD, history of acquired diseases or surgeries.

The exclusion criteria included; type 1diabetes (T1D) which was identified by the onset date of diabetes, and chronic diseases other than DM or CAD. Subjects receiving medications affecting the heart function and/ or insulin were excluded from the study.

### 2.2 Methods

#### 2.2.1 Blood withdrawal and markers assessment

Blood (10 mls) was collected from all 200 subjects after an average of 12 hours fasting. After which it was divided into two portions, the first was collected on vacutainer tubes containing Na fluoride for plasma separation in order to measure fasting plasma glucose (FPG). On the second portion centrifugation was done to separate serum for the measurement of: lipids [TAG, TC, LDLc, high density lipoprotein cholesterol (HDLc)], and the sera levels of miRNAs 9 and 370. Most of the analyses (for the routine work) were done on the day of sample withdrawal, otherwise was stored in a -80°C freezer to complete the rest of the analyses (for measuring circulating miRNAs).

Both FPG and lipids were measured following the manufacturer’s instructions provided by commercially available kits. All measurements were done on Spectro UV photometer by Biochrom. (United Kingdom).

For *RNA extraction and reverse transcription (RT)*: Total RNA was separated from 200 μL of serum using the miRNeasy Mini Kit (Qiagen, Germany) according to instructions of the manufacturer (Qiagen, Germany). Sample to sample variation normalization was done using house-keeping gene as the control/reference gene called (Hs_Snord 68_11) that was measured in each sample following the same steps as miRNAs 9 and 370. The resulting RNA was then dissolved in 50μL of RNase-free water, and stored at −80°C till analysis was done.

Reverse transcription for total RNA was done using miScript II RT kit (Qiagen, Germany) in 4μL miscript Hispec Buffer, 2μL miscript nucleic mix, RNase free water, 2μL miscript reverse transriptase mix, and 12μL RNA template (Qiagen, Germany). For cDNA synthesis, incubation of the RT reaction was done at 37°C for 60 min, and at 95°C for 5 min. Then, the cDNA produced was stored at −20°C till analysis was done.

For *Real-time quantitative PCR* (miscript SYBR Green kit), 5μL of cDNA product was used as template placed in 25μL total reaction volume containing; 12.5μL of Quantitect SYBER Green PCR Master Mix, 2.5 μL of RNase-free water, 2.5μL of miScript Universal Primer, and 2.5μL of miScript Primer Assay. Real-time quantitative PCR was then done using Qiagen rotor gene Q6 Plex real-time PCR system (Qiagen, Germany) at PCR initial activation 95°C for 15 min, followed by 40 cycles of 94° C for 15 s and 55°C for 15 s and 70°C for 30 s. Measurements of miRNAs 9, 370, and (Hs_Snord 68_11) in each sample were carried out on a 96-well plate. Data were analyzed using Rotor gene Q software (Qiagen, Germany), with the automatic setting of Ct. Then, levels of each individual miRNA after normalization to (Hs_Snord 68_11) were calculated by applying the 2-^ΔΔ^Ct method.

#### 2.2.2 Statistical analyses

Results were calculated as mean ± standard error of mean (M ± S.E.M). Normality of distribution was done using Kolmogorov- Smirnov. Significant difference between the studied groups was done using analysis of variance (ANOVA) and post Hoc Tukey was used to detect the least significant difference between individual groups. Pearson χ2 test was used to compare qualitative variables represented as cut off values. For assessing possible cofounders we used general linear model (e.g., age, and BMI). Any skewed data (not normally distributed) were logarithmically transformed before performing linear stepwise regression analyses which were done to study the relation between both miRNAs 9 and 370 (as dependent variables) and the other markers (as the independent variables). Statistical analyses were done using SPSS version 19.0, SPSS Inc, Chicago, IL. P-values less than 0.05, were considered to be significant. Multivariate logistic regression analysis was done to determine the variables that independently contributed to the presence of CAD. Odds ratio and 95% confidence interval were calculated.

## Results

Both demographic and clinical characteristics of the studied groups are shown in S1 Table. Gender was not taken into consideration in the statistical analysis since all patients were above 60 years old; all female subjects were postmenopausal, thus lacking the protective hormonal effect against cardiovascular disease. Logarithmically transformed values were used (Log_10_) for both miRNAs so that the data would be normally distributed for parametric analyses (ANOVA, Spearman's correlation, and multiple linear regression).

**Fig 1:**
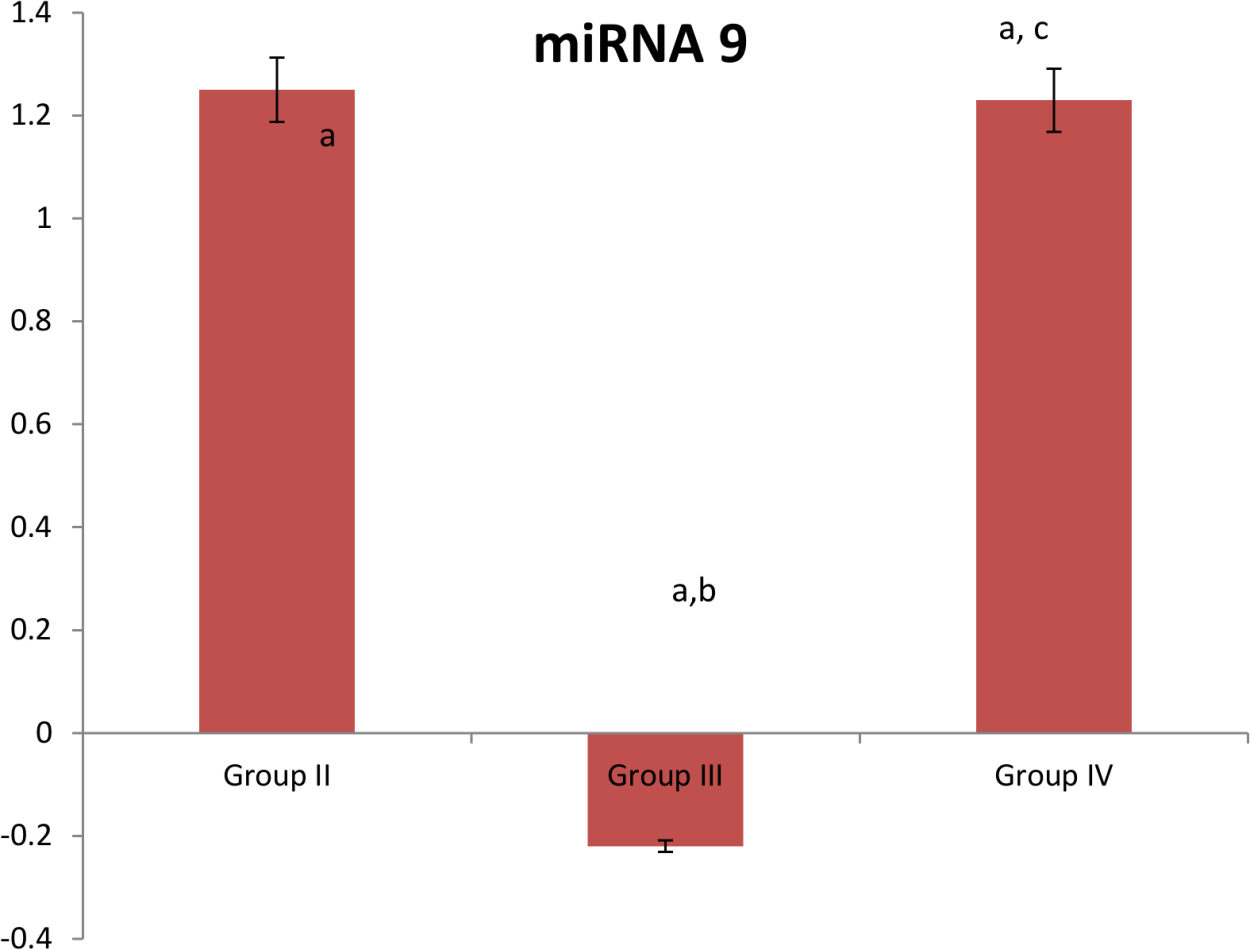
Expression of miRNA 9 in the studied groups. Group II: T2D subjects, Group III: CAD subjects, Group IV: T2D subjects with CAD. Data on the figure is represented as 100% change of control value. a: Significantly different from group I at p = 0.000, b: Significantly different from group II at p = 0.000, c: Significantly different from group III at p = 0.000

Concerning serum miRNA 9 levels, it was found to be significantly elevated in patients with T2D (group II), patients with T2D and CAD (group IV), and significantly decreased in patients with CAD (group III) when compared the control group. In addition, its levels were significantly lower in patients with CAD (group III), while no significant elevation was found in patients with T2D and CAD (group IV) when compared to patients with T2D (group II). In addition, miRNA 9 levels were significantly higher in patients with T2D and CAD (group IV) at p = 0.000 when compared to patients with CAD (group III). On adjusting the effects of age and BMI, the three groups had the same significant difference compared to the control group. [S1 Table, Fig 1]

**Fig 2:**
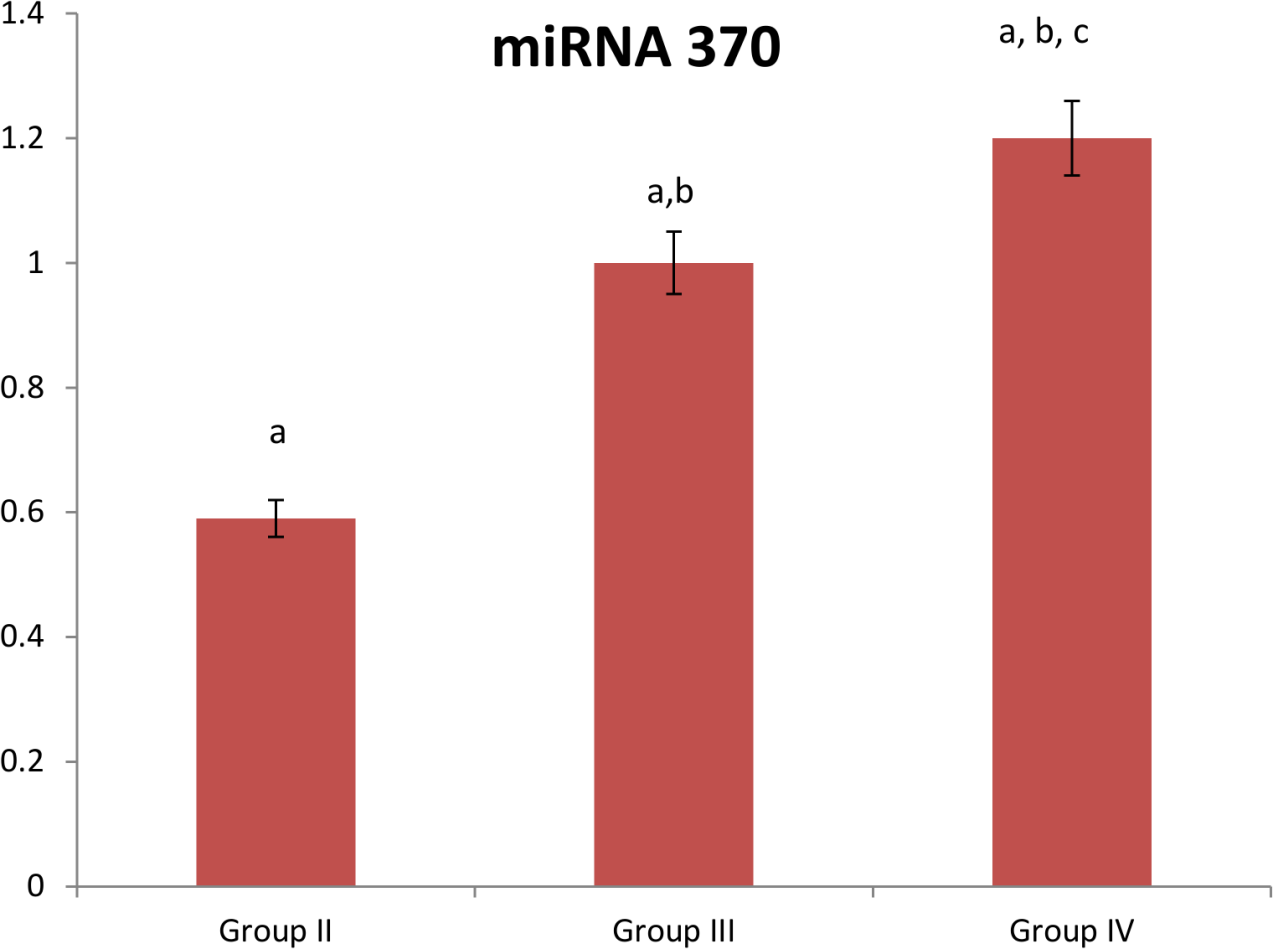
Expression of miRNA 370 in the studied groups. Group II: T2D subjects, Group III: CAD subjects, Group IV: T2D subjects with CAD. Data on the figure is represented as 100% change of control value. a: Significantly different from group I at p = 0.000, b: Significantly different from group II at p = 0.000, c: Significantly different from group III at p = 0.000.

Regarding serum miRNA 370 level, its levels were significantly elevated in patients with T2D (group II), patients with CAD (group III), and patients with T2D and CAD (group IV) than the control group levels at p<0.05. In addition, its levels were significantly elevated in patients with CAD (group III) and patients with T2D and CAD (group IV) when compared to those suffering from T2D (group II). Also, its elevation was significant in patients with T2D and CAD (group IV) at p = 0.000 when compared to patients with CAD (group III). Still after adjusting the effects of age and BMI, patients with T2D and CAD (group IV) showed significant higher levels when compared to other groups. [S1 Table, Fig 2]

On conducting simple linear regression with miRNA 9 as the dependent variable, it was significantly positively associated with other independent variables; BMI, diabetes duration, FPG, TAG, TC, LDLc, Log LDLc/ HDLc ratio and miRNA 370. Mean while, miRNA 9 was significantly negatively associated with HDLc. On using multiple linear stepwise regression analysis using miRNA 9 as dependent variable, only miRNA 370 (β = 0.13, P = 0.02), diabetes duration (β = -0.76, P = 0.001), and BMI (β = -0.24, P = 0.01), remained significantly associated with miRNA 9. [S2 Table]

On applying further analyses on miRNA 9, using multivariate logistic analysis, where miRNA 9 was the dependent variable and the model used contained CAD and log LDLc/ HDLc ratio. Micro RNA 9 was strongly correlated with CAD and log LDLc/ HDLc ratio model at p = 0.000.

On conducting simple linear regression using miRNA 370 as dependent variable, it was significantly positively associated with independent variables including; BMI, diabetes duration, FPG, TAG, TC, LDLc, Log LDLc/ HDLc ratio and miRNA 9. Mean while, miRNA 370 was significantly negatively associated with HDLc. On applying multiple linear stepwise regression analysis using miRNA 370 as dependent variable, only miRNA 9 (β = 0.19, P = 0.02), HDLc (β = -0.44, P = 0.001), and TC (β = 0.26, P = 0.01) and remained significantly associated with miRNA 370. [S2 Table]

On applying Chi square analysis in T2D patients (group II), miRNA 9 showed a cut off value of 8 folds with sensitivity 72% and specificity 82%, when compared to patients with T2D and CAD (group IV) as positive control. In case of miRNA 370 chi square analysis calculated a cut off value of 6 folds with sensitivity 26% and specificity 82% sensitivity, when compared to patients with T2D and CAD (group IV) as positive control. When the sensitivity and specificity were combined for both miRNAs it increased to reach 84% in patients with T2D (group II) when compared to patients with T2D and CAD (group IV) as positive control. [S3 Table]

Meanwhile, on applying Chi square analysis in patients with CAD (group III), miRNA 9 showed a cut off value of 8 folds with sensitivity 6% and specificity 82% when compared to patients with T2D and CAD (group IV) as positive control. In case of miRNA 370, chi square analysis calculated a cut off value of 6 folds showing sensitivity 72% and specificity 82% when compared to patients with T2D and CAD (group IV) as positive control. Also, when the sensitivity and specificity were combined for both miRNAs in patients with CAD (group III) showed no significant increase when compared to patients with T2D and CAD (group IV) as positive control. [S4 Table]

## Discussion

Type 2 diabetes mellitus is one of the main risk factors for the incidence of CAD due to hyperglycemia induced endothelial dysfunction [18]. Altered regulation of both miRNA levels and function have been associated with several conditions, including CAD [19].Therefore this study was conducted to evaluate the association between two miRNAs; 9 and 370 in T2D patients with/without CAD.

The current study correlated miRNA 9 with hyperglycemia in human sera where its levels were found to be significantly elevated in T2D with and without CAD, and its levels were lower in those suffering from CAD when compared to control healthy patient. Elevated miRNA 9 levels in patients with T2D and its positive correlation with FPG could be explained as miRNA 9 targets Onecut 2 a transcription factor[20], which decreases Granuphilin (a negative regulatory element of the exocytotic process of insulin) [21], [22]. Thus, increased levels of miRNA9 decreases glucose stimulated insulin secretion, which is concomitant with our findings.

To our knowledge, this is the first study done to correlate miRNA 9 with lipid profile markers in human sera, no data was available to contradict or concur with our results. Where, sera miRNA 9 levels were positively correlated with BMI, TAG, TC, LDLc, and LDLC/HDLc ratio in patients with T2D with or without CAD while negatively correlated with that of HDLc in all T2D, especially in those suffering from with T2D and CAD. Another interesting finding in this study was that miRNA 9 remained positively correlated with BMI even after conducting multiple linear regression analysis. Risk factors for the development of T2D and CAD complication of DM include age, gender, dyslipidemia, increased blood pressure, genetic predisposition of DM, [18], and impaired insulin secretion on which miRNA 9 acts [20].

As for sera miRNA 370, its levels were significantly increased in patients with T2D and CAD, CAD only, and patients with T2D. This was also found in another study that showed the use of certain miRNA imprints including miRNA 370, in the screening of patients at risk for developing acute coronary disease [22].

In addition, in the current study miRNA 370 showed positive association with BMI, FPG, TAG, TC, LDLc, and LDLc/HDLc ratio, while negative association with HDLc. Several miRNAs including miRNA 370 were found to be associated with lipid metabolism regulation [21]. Micro RNA 370 reduces β-oxidation of fatty acids but on the other hand increases the expression of another miRNA that results in increasing cholesterol synthesis and β-oxidation of fatty acids, thus promoting insulin resistance leading to T2D and CAD complication of DM [16]. This explains the obtained observations of positive correlation between miRNA370 and dyslipidemia in patients with T2D with CAD, and was also found in another study, where miRNA370 levels were increased in patients with hyperlipidemia and their expression correlated with CAD incidence [23]. Changing miRNA 370 levels in human hepatocytes resulted in changing β-oxidation of fatty acids and synthesis of TAG, which was linked to changes in the levels of fatty acid synthase and acetyl-CoA carboxylase [16].

In addition on using multiple linear regression analysis miRNA 370 remained positively associated with TC while negatively correlated with HDLc. Thus increased expression of miRNA 370 combined with increased TC and decreased HDLc represent essential constituents for the occurrence and vulnerability of atherosclerosis [24].

Another interesting finding in the current study is the positive correlation found between miRNA 9 and 370 on conducting simple linear regression analysis. Mean while, when the effect of other parameters was included by conducting multiple linear regression, this positive correlation still remained. In addition, the sensitivity and specificity of both miRNAs were calculated in both patients with T2D (group II) and patients with CAD (group III) using patients with T2D and CAD (group IV) as the positive control, miRNA 9 and miRNA 370 showed 72% and 28% sensitivity, respectively, in patients with T2D (group II). While miRNA 9 and miRNA 370 showed 6% and 72% sensitivity, respectively, in patients with CAD (group III). On combining both miRNAs the sensitivity and specificity in patients with T2D (group II) increased reaching 84% instead of 76% compared to using miRNA 9 only in this group.

Another novel finding, on conducting multivariate logistic regression analysis using only miRNA 9 as the dependent variable and CAD with log LDLc/HDLc as the main effect model, there was a strong correlation between them reaching p = 0.000. This gives additional evidence to our novel association of miRNA 9 with the development CAD on top of T2D.

In conclusion, in this study the association of dyslipidemia and miRNAs in T2D and CAD has been demonstrated, where positive correlation between the two miRNAs (9 and 370) with FPG, and dyslipidemia in the sera of both T2D and CAD patients implied their role as associative markers in the diagnosis and prognosis of CAD complication of T2D. Accordingly, miRNAs 9 and 370 could be potential markers recommended for the clinical use in the prognosis and follow up of patients with T2D at risk to develop CAD. Further clinical studies using larger sample size should be done, to support the previous findings.

## Supporting Information

S1 Table Demographic and clinical characteristics of the studied groups. (DOCX)

S2 Table Simple linear regression of miRNAs 9 and 370 as dependant variables. (DOCX)

S3 Table Sensitivity and specificity of miRNA9 and miRNA 370 in patients with T2D (group II). (DOCX)

S4 Table Sensitivity and specificity of miRNA9 and miRNA 370 in patients with CAD (group III). (DOCX)
