# Supplementary material for: MicroRNAs 9 and 370 Association with Biochemical Markers in T2D and CAD Complication of T2D
Source: PLoS One. 2015 May 15;10(5):e0126957. doi: 10.1371/journal.pone.0126957 (PMC4433316; doi:10.1371/journal.pone.0126957)
Supplement: S1 Table — (DOCX) [file pone.0126957.s001.docx]

**Supporting information**

**This is the S1 Table 1 title: Demographic and clinical characteristics of the studied groups**

| **Groups/**  **Parameters** | **Group(I) Control (n=50)** | **Group(II)**  **T2D (n=50)** | **Group(III)**  **CAD (n=50)** | **Group(IV)**  **T2D with CAD (n=50)** |
| --- | --- | --- | --- | --- |
| Age (Years) | 62.22±0.69 | 62.06±1.26 | 62.32±0.56 | 62.30±0.45 |
| BMI (Kg/m^2^) | 23.82±0.13 | 27.58±0.27^a^ | 27.88±0.23^a^ | 28.87±0.32^a,b^ |
| Male n (%) | 36 (72%) | 32 (64%) | 38 (76%) | 35 (70%) |
| Female n (%) | 14 (28%) | 18 (36%) | 12 (24%) | 15 (30%) |
| Diabetes duration (Years) | --- | 12.22±0.30 | --- | 12.06±**0.30** |
| Coronary artery disease  Angina n (%)  Clots n (%)  Ischemia n (%) | ---- | ---- | Angina 30 (60%)  Clots 12 (24%)  Ischemia 8 (16%) | Angina 28 (56%)  Clots 10 (20%)  Ischemia 12 (24%) |
| FPG(mg/dl) | 88.26±1.37 | 185.88±5.89 ^a^ | 94.38±0.96^b^ | 171.16±2.39^a, b,c^ |
| TAG(mg/ dL) | 140.64±2.62 | 171.96±5.50^a^ | 177.40±3.53^a^ | 195.58±3.66^a, b, c^ |
| TC(mg/dL) | 155.52±1.44 | 188.48±4.20^a^ | 190.52±2.34^a^ | 200.42±2.56^a, b^ |
| HDLc(mg/dL) | 61.72±0.70 | 46.40±1.05^a^ | 41.68±0.67^a,b^ | 39.10±0.65^a,b^ |
| LDLc(mg/ dL) | 57.44±0.62 | 107.96±5.60^a^ | 91.16±2.23 ^a,b^ | 105.06±2.90^a,c^ |
| LDLc/HDLc ratio**^@^** | -0.03±0.006 | 0.33±0.01^a^ | 0.34±0.01^a^ | 0.42±0.01^a,b,c^ |
| miRNA 9 **^@^** (expression) | 0.00±0.00 | 1.15±0.04^a^ | -0.21±0.10 ^a,b^ | 1.22±0.09 ^a,c^ |
| miRNA 9**^@^**#(expression) | 0.00±0.00 | 1.18±0.07^a^ | -0.17±0.07 ^a, b^ | 1.31±0.08^a,c^ |
| miRNA 370 **^@^** (expression) | 0.00±0.00 | 0.58±0.07^a^ | 0.98±0.04^a, b^ | 1.16±0.06^,a, b, c^ |
| miRNA 370 **^@^**#(expression) | 0.00±0.00 | 0.59±0.05^a^ | 1.00±0.05^a,b^ | 1.20±0.06^a,b,c^ |

This is the S1 table (1) legend: Values are expressed in terms of (Mean ± SEM). BMI, body mass index; T2D, type 2 diabetes mellitus; FPG fasting plasma glucose; TAG triglycerides; TC total cholesterol; HDLc high density lipoprotein cholesterol, and LDLc low density lipoprotein cholesterol.

a : Significantly different from control group I at p=0.000

b: Significantly different from group II at p=0.000

c: Significantly different from group III at p=0.000

^@^: Log transformed values were used.

#: Mean ± SEM by general linear model with adjustment of age and BMI.
