# Supplementary material for: MicroRNAs 9 and 370 Association with Biochemical Markers in T2D and CAD Complication of T2D
Source: PLoS One. 2015 May 15;10(5):e0126957. doi: 10.1371/journal.pone.0126957 (PMC4433316; doi:10.1371/journal.pone.0126957)
Supplement: S2 Table — (DOCX) [file pone.0126957.s002.docx]

**Supporting information**

**This is the S2 Table 2 title: Simple linear regression of miRNAs 9 and 370 as dependant variables**

| **variable** | **MiRNA 9** | | **MiRNA 370** | |
| --- | --- | --- | --- | --- |
|  | **β (r)** | **P** | **β (r)** | **P** |
| **Age** | 0.044 | 0.537 | -0.004 | 0.953 |
| **BMI** | 0.335** | 0.00 | 0.536** | 0.000 |
| **Diabetes durat.** | 0.775** | 0.000 | 0.314** | 0.000 |
| **FPG** | 0.591** | 0.000 | 0.387** | 0.000 |
| **TAG** | 0.331** | 0.000 | 0.550** | 0.000 |
| **TC** | 0.331** | 0.000 | 0.508** | 0.000 |
| **HDLc** | -0.305** | 0.000 | -0.591** | 0.000 |
| **LDLc** | 0.451** | 0.000 | 0.505** | 0.000 |
| **LDLc/HDLc ratio^@^** | 0.411** | 0.000 | 0.576** | 0.000 |
| **miRNA 9** | --- | --- | 0.343 ** | 0.000 |
| **miRNA 370** | 0.343** | 0.000 | --- | --- |

This is the S2 table (2) legend:

*: Significant at p<0.05

** Significant at p<0.01

^@^: Log transformed values were used. BMI, body mass index; FPG fasting plasma glucose; TAG triglycerides; TC total cholesterol; HDLc high density lipoprotein cholesterol; LDLc low density lipoprotein cholesterol.
