# Supplementary material for: MicroRNAs 9 and 370 Association with Biochemical Markers in T2D and CAD Complication of T2D
Source: PLoS One. 2015 May 15;10(5):e0126957. doi: 10.1371/journal.pone.0126957 (PMC4433316; doi:10.1371/journal.pone.0126957)
Supplement: S4 Table — (DOCX) [file pone.0126957.s004.docx]

**Supporting information**

**This is the S4 Table 4 title: Sensitivity and specificity of miRNA9 and miRNA 370 in patients with CAD (group III)**

| **miRNA** | **Sensitivity** | **Specificity** | **Combined** |
| --- | --- | --- | --- |
| **miRNA 9** | **6%** | **82%** | **72%** |
| **miRNA 370** | **72%** | **82%** |  |
